# Selective depletion of a minor subpopulation of B-chronic lymphocytic leukemia cells is followed by a delayed but progressive loss of bulk tumor cells and disease regression

**DOI:** 10.1186/1476-4598-8-106

**Published:** 2009-11-18

**Authors:** Aaron E Foster, Fatma V Okur, Ettore Biagi, An Lu, Gianpietro Dotti, Eric Yvon, Barbara Savoldo, George Carrum, Michael Andreeff, Margaret A Goodell, Helen E Heslop, Malcolm K Brenner

**Affiliations:** 1 Center for Cell and Gene Therapy, Baylor College of Medicine, The Methodist Hospital and Texas Children's Hospital, Houston, TX, 77030 USA; 2 Department of Molecular Hematology/Therapy, MD Anderson Cancer Center, Houston, TX, United States, 77030 USA

## Abstract

Cancer precursor/progenitor cells may initiate and sustain the growth of tumors, but evidence for their existence in human disease is indirect, relying on their *in vitro *properties and animal models. More directly, specific elimination of these rare cells from cancer patients should produce a delayed but progressive disappearance of differentiated malignant progeny. Here, we describe selective eradication of a putative precursor population in a patient with B-cell chronic lymphocytic leukemia, followed 6 months later by a progressive loss of mature tumor cells without further treatment. This outcome supports the presence of a rare population of precursor/progenitor cells in human malignancies, and suggests benefit from their removal.

## Findings

Tumors may contain a distinct minority population of "stem cells", but to date only indirect evidence exists that such cells initiate or sustain human cancers [1,2]. The putative stem cell population has been distinguished by a particular surface phenotype, which may be unique to a given tumor type, or by certain functional properties, such as the ability to efflux Hoechst dyes, which produces a distinct side-population (SP) of cells on fluorescent analysis [3]. Irrespective of their distinguishing characteristics, these subpopulations exhibit asymmetric cell division, enhanced proliferation and greater facility to form tumors in mice [4-9]. Although suggestive, none of these criteria are definitive of a cancer stem cell, and analysis may be confounded by artifacts of the system used for study [9]. Moreover, some tumor cells, such as those from B cell chronic lymphocytic leukemia (B-CLL) grow poorly *in vitro *and in animal models, further hampering identification of a true stem cell population. It is not possible to identify a human tumor stem cell population with absolute rigor, since this would require demonstration that only the putative stem cell population could induce disease when administered to human subjects. It is, however, feasible to attempt to demonstrate the reverse, and show how selective removal of a putative stem cell population is followed, after a delay, by the subsequent and progressive loss of the bulk population which is unable to sustain itself past the life span of the "committed" tumor cells. We now describe how administration of a B-CLL tumor cell vaccine generated a transient immune response that selectively and completely removed a putative precursor population, identified by SP analysis, in B-CLL cells from peripheral blood, but had no initial effect on lymphosplenomegaly or total (non-SP) B-CLL cell counts. Continued follow-up over the following 18 months, however, revealed a progressive and continuing reduction in the bulk (non-SP) peripheral blood B-CLL count and in lymphosplenomegaly that began 6 months after completion of immunization and continued progressively over the next 12 months.

At the time of study entry, P1300 was a 65 old male with Stage IV B-CLL, diagnosed 2 years previously. He had received no treatment before vaccination. Pre-vaccination staging showed multiple enlarged lymph nodes by CT scan in the neck, axilla, mediastinum abdomen and inguinal region, splenomegaly (20 × 18 × 8 cm), WBC (31,000/μL), hemoglobin (12.7 gm/dL), platelets (84,000/μL), β2 macroglobulin (4.4 mg/mL), reticulocyte count (1.0%), LDH (211 U/L), diffuse lymphoid infiltrate consistent with B-CLL on bone marrow biopsy, and deletion of 11q and 13q on FISH. After informed consent, he was immunized with CD40L and IL-2 gene-modified, irradiated autologous B-CLL cells on a RAC-NIH, FDA and IRB approved protocol [10]. Briefly, PBMC (>90% CD5/CD19/CD20) were harvested from P1300 and co-cultured on MRC-5 (a human embryonic lung fibroblast cell line; ATCC) transduced with human CD40L and interleukin-2 (IL-2) as previously described [10]. After confirmation of transduction by flow cytometry for hCD40L (94%) and IL-2 secretion (2,412 pg/ml/10^6 ^leukemic cells), the gene-modified tumor vaccine was irradiated (30 Gy) and cryopreserved. P1300 subsequently received 6 subcutaneous injections on weeks 0, 1, 2, 6, 8 and 10.

**Figure 1:**
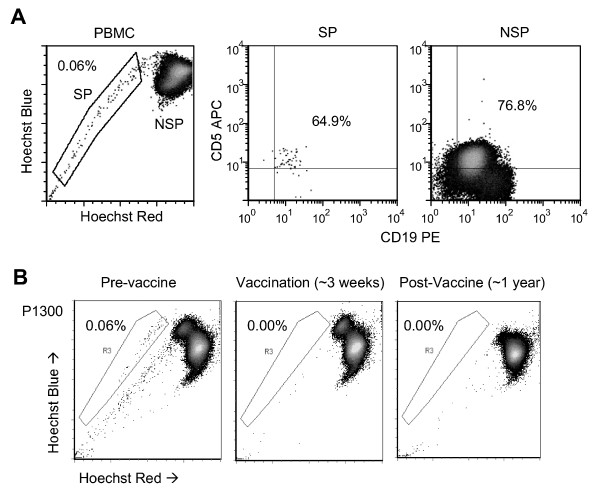
**A distinct CD5^+^CD19^+ ^SP phenotype is present in B-CLL patient peripheral blood**. (**A**) PBMC collected from B-CLL patient P1300 were stained with Hoechst 33342 dye followed by antibody and propidium iodide labeling and subsequent analysis by flow cytometry. Cells were gated on either SP or NSP and examined for expression of CD5 and CD19 showing both populations are positive for CD5 and CD19. (**B**) CD5^+^CD19^+ ^SP frequency was analyzed longitudinally from PBMC samples collected prior to CD40L/IL-2 autologous B-CLL vaccination (left panel), during immunization (middle panel) and after immunization (right panel), demonstrating the elimination of B-CLL SP cells during treatment.

**Figure 2:**
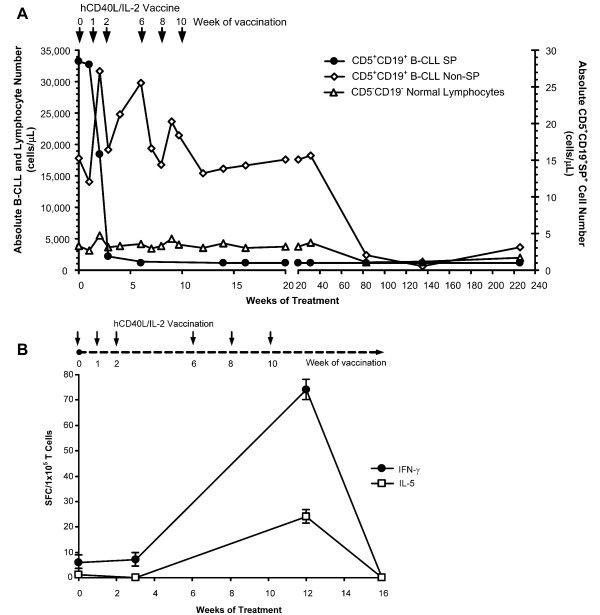
**B-CLL-specific immunity associated with elimination of B-CLL SP cells and subsequent decrease in leukemic cell counts in one patient treated with hCD40L/IL-2**. (**A**) Vaccination with hCD40L/IL-2 gene-modified tumor cells resulted in the stable elimination of SP cells in the peripheral blood of one patient (P1300), in whom SP cells did not return when immunization was complete. Analysis of leukemic cell number in the peripheral blood of patient P1300 showed a delayed decline in circulating B-CLL cells, beginning six months after loss of circulating SP cells. Absolute cell number was calculated from the white blood cell (WBC) count multiplied by the percentage of B-CLL (CD5^+^CD19^+^), normal B cell (CD5^-^CD19^+^) and T cells (CD3^+^), and SP (CD5^+^CD19^+^SP^+^) as determined by flow cytometry. Arrows indicate when patient received vaccination (6 vaccine injections total at 0, 1, 2, 6, 8 and 10 weeks). (**B**) P1300 received 6 subcutaneous injections of hCD40L/IL-2 gene-modified autologous tumor cells at 0, 1, 2, 6, 8 and 10 weeks (indicated by arrows). B-CLL-specific immunity was measured by selecting CD4 and CD8 T cells from PBMC by magnetic separation and measuring IFN-γ and IL-5 by ELIspot following a 36 hour co-culture with autologous tumor cells (SFC; spot-forming cells per 1 × 10^5 ^T cells). ELIspot analysis was performed on weeks 0 (pre-vaccine), 3, 12 and 16 (two months after last vaccination).

Prior to vaccination, PBMC from P1300 were labeled with Hoechst 33342 as previously described [3], and co-stained with CD5 and CD19 antibodies to discriminate tumor cells from normal B lymphocytes. Flow cytometry detected a distinct CD5^+^CD19^+ ^SP phenotype (Figure 1a). We excluded the possibility of non-specific staining by Verapamil inhibition, and confirmed that CD5^+^CD19^+ ^SP cells are restricted to leukemic patients by examining the peripheral blood from healthy donors, where 0/5 contained SP cells (not shown). We next followed the CD5^+^CD19^+ ^SP cells in P1300's PBMC during (weeks 3 and 6) and after vaccination (10 weeks to 2.5 years post-immunization). The distinct SP population present prior to immunization diminished and then disappeared during immunization (Figure 1b) even though no equivalent change was initially observed in total B-CLL counts (Figure 2a). During vaccination, the T cell immune response to autologous B-CLL tumor cells (as measured by the frequency of IFN-γ and IL-5 ELIspots) was markedly increased (Figure 2b). However, this immune response diminished after immunization, returning to baseline 6 weeks after the last vaccination. The SP cells, however, did not return. Hence, a detectable B-CLL-specific T cell immune response was associated with the observed decrease in CD5^+^CD19^+ ^SP cells in the peripheral blood of the patient. The loss of SP cells following immunization was selective, since there was no overall disease response to the vaccine during initial follow-up (Figure 2a). After 6 months, however, this patient began to show a progressive decline in peripheral blood B-CLL leukemic cells, which fell from 17,794 cells/μL to 639 cells/μL by week 135 (Figure 2a). This reduction was associated with a progressive reduction of lymphadenopathy and splenomegaly, and normalization of platelet numbers, leading to a reduction of his Rai Stage from 4 to 0, an effect observed in the absence of a continued immune response and without further treatment.

We propose that the selective removal of a putative tumor precursor cell population has resulted in the delayed disappearance of the more "mature" malignant cells from the host, as the pipeline of tumor development became interrupted. Our *in vivo *clinical data offer support to a substantial body of existing pre-clinical analyses of SP cells that have also suggested that this functional phenotype identifies a precursor cell population for both normal hematopoiesis and other malignancies [3,11-18]. While SP analysis uses enhanced export of Hoechst dyes to identify the precursor population, this ABC-transporter mediated activity [19,20] can also reduce the intracellular concentrations, and thus the effectiveness, of many commonly used chemotherapeutic drugs [11-13]. Hence, tumor SP cells may therefore both be a tumor precursor population and have the ability to resist common chemotherapeutic agents, contributing to disease persistence or relapse. But although B-CLL SP cells may have greater drug resistance than non-SP cells, our data show that they may also be more sensitive to control by immune T cells than the bulk population. Kipps' group, who first reported that expression of transgenic murine CD40L on autologous B-CLL cells could induce an anti-tumor response after intravenous infusion [21], have subsequently shown that vaccinated patients develop an antibody response towards ROR1, a tyrosine kinase receptor involved in recognition of Wnt5a and involved in cell motility and asymmetric cell division, characteristics associated with stem cell behavior [22,23]. While the effects of such immunization on the malignant SP cells in this study are unknown, we hypothesize that differential antigen expression by B-CLL SP cells may allow selective elimination by the immune system. Here, CD40 activation of autologous B-CLL tumor cells may upregulate tumor-associated antigens, such as survivin or RHAMM (receptor for hyaluronic acid-mediated motility) [24,25] which may be expressed *in vivo *by B-CLL SP cells, resulting in their depletion following immunization.

In summary, we conclude that circulating B-CLL cells contain at least two distinct populations, SP and non-SP, and that selective removal of the SP component cells can be followed by the delayed disappearance of non-SP in the absence of further treatment or a continuing immune response. These data support the existence of a true tumor precursor population in this human malignancy and suggest benefit from their removal.

## Abbreviations

B-CLL: B cell chronic lymphocytic leukemia; SP: side-population; PBMC: peripheral blood mononuclear cells; ELIspot: enzyme-linked immunosorbent spot assay.

## Competing interests

The authors declare that they have no competing interests.

## Authors' contributions

AEF, FVO and MKB designed and performed research, analyzed data and wrote the paper. EB, EY and AL analyzed data. GD, BS, and MAG contributed to research analysis and discussion. GC, MA and HEH contributed to patient care. All authors read and approved the final manuscript.
